# The Effects of 0.01% Atropine on Adult Myopes’ Contrast Sensitivity

**DOI:** 10.3389/fnins.2021.624472

**Published:** 2021-02-19

**Authors:** Ziyun Cheng, Jianhui Mei, Suqi Cao, Ran Zhang, Jiawei Zhou, Yuwen Wang

**Affiliations:** State Key Laboratory of Ophthalmology, Optometry and Vision Science, School of Ophthalmology and Optometry, Affiliated Eye Hospital, Wenzhou Medical University, Wenzhou, China

**Keywords:** atropine, contrast sensitivity, myopia control, visual perception, myopia

## Abstract

**Purpose:**

Atropine at a low concentration is considered a safe and effective treatment to mitigate myopia progression. However, the potential unwanted side effects of administering atropine at a low dose on visual functions other than best corrected visual acuity has not been investigated. In this study, we investigate the short-term (12,16, and 20 h) and long-term (1, 2, and 4 weeks) effects of 0.01% atropine (i.e., 0.1 mg/ml) on contrast sensitivity (CS) in patients with myopia.

**Methods:**

Thirty adults (23.33 ± 2.93 years old) with myopia between -1.00 and -6.00 diopters (D), astigmatism of -1.50 D or less, and anisometropia of 1.00 D or less, participated in this prospective, masked, placebo-controlled, randomized study. The participants were randomly assigned to receive 0.01% atropine or polyvinyl alcohol eye drops once nightly to both eyes for four weeks. CS was measured binocularly at baseline and 12, 16, 20 h, 1, 2, and 4 weeks after the first use of the eye drops.

**Results:**

There was no statistically significant differences of CS found between atropine and placebo-controlled groups in both short-term and long-term. There was no statistically significant interaction effect found between the time and group.

**Conclusion:**

We demonstrated no significant deleterious effect of 0.01% atropine on adult myopes’ CS.

## Introduction

The prevalence of myopia has increased sharply over the last several decades (Holden et al., 2016; Hopf and Pfeiffer, 2017; Grzybowski et al., 2020). Approximately 28.3% of the world’s population was myopic in 2017 (Hopf and Pfeiffer, 2017), and the rate is expected to reach 40% by 2030 (Holden et al., 2016). At the same time, the prevalence of high myopia rose dramatically from 2.7 to 5.2% between 2000 and 2020, with associated concomitant surge of sight-threatening complications (Holden et al., 2016; Ziemssen et al., 2017). A variety of clinical interventions, including multifocal spectacles, contact lenses, and pharmaceutical agents, have been put into practice trying to slow down the rapid progression of myopia. Recent studies have suggested that topical atropine might be the most effective method (Huang et al., 2016; Walline et al., 2020a). Though efficacy of administration of atropine at a low concentration (e.g., 0.01%) is weaker than high concentration (e.g., 1, 0.5%), it has fewer side effects (e.g., photophobia, blurry near vision and allergic conjunctivitis) and minimum rebound after drop cessation (Chua et al., 2006; Chia et al., 2012; Chia et al., 2014; Chia et al., 2016; Yam et al., 2019; Yam et al., 2020). Thus, there is a preference for 0.01% atropine among pediatric ophthalmologists in myopia control (Zloto et al., 2018; Wu et al., 2019).

One side effect is the effect of myopic control interventions on patient’s visual perception. The application of low-concentration atropine (e.g., 0.01, 0.05%) has been shown to less likely impair myopes’ best corrected visual acuity (VA) (Chia et al., 2012; Moon and Shin, 2018; Yam et al., 2019; Yam et al., 2020). For example, Chia et al. (2012) applied 0.5, 0.1, and 0.01% atropine respectively to three groups of subjects once nightly to both eyes for 2 year and found mean best-corrected distant VA was not significantly affected by atropine use; Yam et al. (2019) administered similar protocol as Chia et al. (2012) (i.e., eye drops once nightly to both eyes) with low-concentration atropine eye drops at 0.05, 0.025, and 0.01% and placebo for a year and demonstrated VA was not significantly influenced in each group. However, VA, as one of the most important visual functions, only describes patients’ visual performance in recognizing high contrast letters. A minimal to no change of VA does not mean that patients’ perception on low contrast targets is intact (Joltikov et al., 2017; Zhao et al., 2017; Alahmadi et al., 2018). Moreover, there is evidence that myopic control interventions, such as wearing multifocal spectacles or contact lenses, reduce myopes’ low-contrast vision acuity compared with single vision lens (Lu et al., 2020; Walline et al., 2020b). Therefore, it is necessary to further assess the effect of low-concentration atropine on myopes’ other visual functions before widespread application in clinical practice.

Contrast sensitivity function (CSF) provides a much more comprehensive assessment of spatial vision at different contrast conditions and a variety of spatial frequencies and has been used to evaluate and screen a variety of visual disorders (Chatzistefanou et al., 2005; Joltikov et al., 2017; Alahmadi et al., 2018). In animal models, it has been found that one drop of 1% atropine can actually increase contrast sensitivity (CS), at least, at low spatial frequency (e.g., 0.03 and 0.20 cycles per degree) in mice and chicks, measured by optomotor paradigm (Diether and Schaeffel, 1999; Schmucker and Schaeffel, 2006). In the mice, the contrast threshold of 0.03 cycles per degree was down to 16% from 24% (Schmucker and Schaeffel, 2006). Although there has been evidence that sustained penalization of the fellow eye with atropine is one of the standard treatments for amblyopia, improving their CS after the therapy (Menon et al., 2008; DeSantis, 2014), there has been, to our best knowledge, no study about the effects of atropine on myopes’ CS. Previous experiments demonstrate that best corrected VA of myopia is not affected after the application of low-concentration atropine (Chia et al., 2016; Yam et al., 2019; Yam et al., 2020). In the current study, we are interested to know whether atropine could influence myopes’ CS. To address this, we have applied 0.01% atropine or polyvinyl alcohol in two groups of myopes for four weeks and measured observers’ CS before and at 12, 16, 20 h and 1, 2, 4 weeks after the first use of the eye drops. We have evaluated both short-term (e.g., within one day) and long-term (e.g., weeks) influences of low-concentration atropine application on myopes’ visual perception.

## Materials and Methods

### Participants

Thirty adults (23.33 ± 2.93 years old; 18 females) with myopic refraction between –1.0 D and –6.0 D, astigmatism of less than 1.5 diopters (D) in both eyes, and anisometropia of less than 1.0 D were enrolled. All participants have normal or corrected-to-normal vision (logMAR acuity ≤0.00). Excluded were those with ocular pathology (e.g., amblyopia, strabismus, glaucoma, conjunctivitis), ocular surgery history, abnormal binocular function, allergy to atropine, systemic ill health (e.g., diabetes or autoimmune diseases), or previous use of atropine or orthokeratology. All participants were naive to the purpose of the study. The study and protocol conformed to the tenets of the Declaration of Helsinki and was approved by the Ethics Committee of Eye hospital of Wenzhou Medical University. Written informed constant was obtained from each participant.

### Design

**FIGURE 1:**
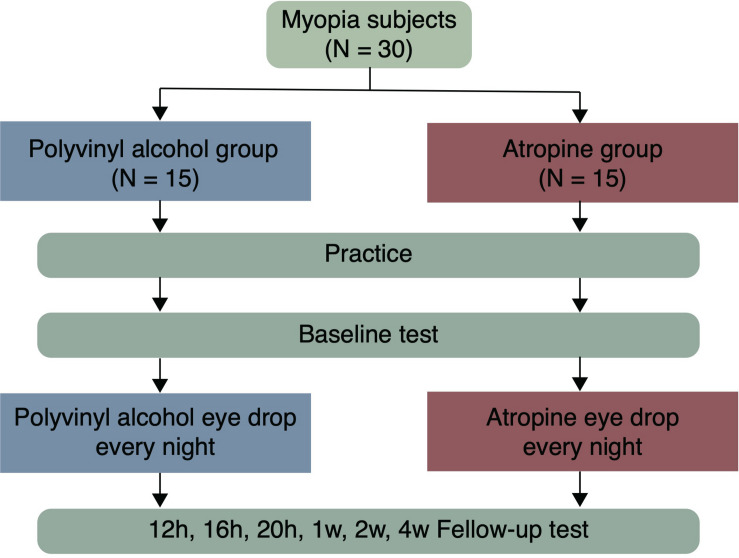
A flow chart illustrating the study procedure.

Participants were allocated randomly to the atropine or the placebo-control group according to a computer-generated randomization list, and respectively received one drop of 0.01% atropine (0.04 mg/0.4 ml unit-concentration, preservative free, Shengyang Xinqi Eye Hospital Co., Ltd.) or polyvinyl alcohol (0.5 ml unit-concentration, preservative free, Xindongshengji Co., Ltd., Taiwan) once nightly (after 8:00 pm) in both eyes for 4 weeks. The eye drops were given by the authors (Z Cheng and J Mei), and the participants were unaware of which eye drop was given. CS was binocularly measured with individual’s optimal spectacle correction in his/her each visit. Before the baseline test in the first visit, participants had been given a chance to be familiarized with the test. Follow-up visits were then scheduled after 12, 16, 20 h, 1, 2, and 4 weeks from their first use of the eye drops (Figure 1).

### Apparatus

All tests were performed on a visual function test workstation (Zhishiyuan, JH-P02, Model NO.102JST190828001, Jiangsu Juehua Medical Technology Co., Ltd.) in a dark room. The workstation consists of a PC and a display. Stimuli were generated and controlled by the PC, and presented on the display. The display was GAMMA-corrected, has a resolution of 2560 x 1440 pixels, a refresh rate of 60 Hz, and an average luminance of 74.5 cd/m^2^.

### Study Procedures

**FIGURE 2:**
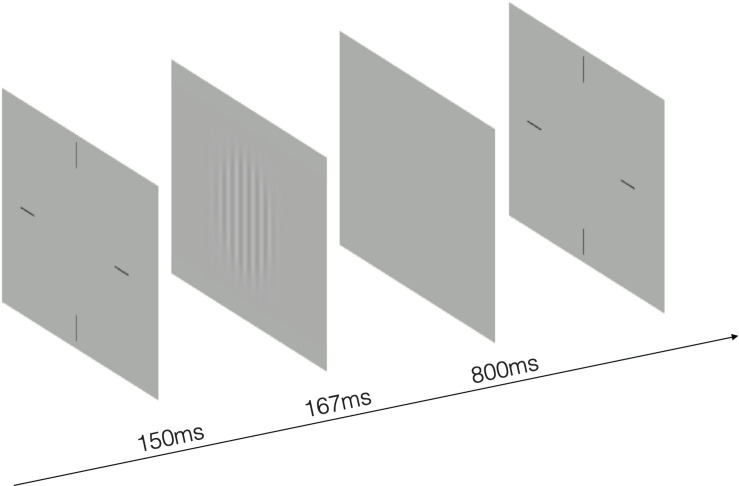
An illustration of the test. The stimulus was a sinusoidal grating of vertical or horizontal orientation with spatial frequency of 1.5, 3, 6, 12, 18, 24 cycles per degree. A 0.5-degree Gaussian ramp was added around the stimulus to reduce the edge effect.

The stimulus was a sinusoidal grating with spatial frequency of 1.5, 3, 6, 12, 18, 24 cycles per degree (cpd) and subtended 3.0° × 3.0° at a viewing distance of 2 m. In order to reduce the edge effect, 0.5-degree Gaussian ramp was added around the stimulus. Before the start of the test, there was an introduction about the entire experimental process, stimuli and task. A brief beep prompted the start of the trial, together with presentation of a crosshair (3.0 × 3.0°) to indicate the location of the stimulus. After 150 ms, the cross disappeared and stimulus grating of vertical or horizontal orientation (with equal probability) were displayed for 167 ms. A blank background with mean luminance (74.5 cd/m^2^) was then displayed and participants were asked to the orientation with corresponding arrow key in the keyboard. Inter-trial interval was 800 ms (Figure 2). A Psi method (Kontsevich and Tyler, 1999) controlled the grating contrast and estimated contrast threshold that corresponds to 80.3% correct for each spatial frequency, separately. CS was calculated as the reciprocal of contrast threshold. There were 270 trials in total, with 45 trials in each spatial frequency. The work station adopted the bit-stealing method (Tyler, 1997) to achieve high-precision gray-scale stimulation.

### Data Analysis

A Shapiro–Wilks test was performed on each dataset to evaluate normality of our dataset. The demographic characteristics (e.g., age, gender, and refractive error) between the two treatment groups were evaluated by two-sample *t*-test for normality dataset, Wilcoxon rank sum test for non-normally distributed dataset, and Chi-square test for qualitative dataset. A one-way analysis of variance (ANOVA) was used to compare the CSF at baseline between groups. A repeated-measure ANOVA was used to evaluate the main effects of group, time and the interaction between them after treatment. The area under the log CSF curve (AULCSF), which is a widely used summary metric of the CSF function (Joltikov et al., 2017; Alahmadi et al., 2018; Zheng et al., 2018), was calculated by using the trapezoid method with CS in logarithmic values at 1.5–24 cpd (Rosén et al., 2014), and compared between groups and follow-up time sessions. *P* value <0.05 was considered statistically significant. Statistical analyses were performed using SPSS 26.0 software (IBM Crop., Armonk, NY. Released 2019).

## Results

At the initial pretreatment visit, there were no significant differences between the groups in mean age, gender, and refractive error (mean age, *P* = 0.407; gender, *P* = 1.000; refractive error: OD, *P* = 0.512; OS, *P* = 0.880). The individual information of participants was exhibited in Table 1. One-way ANOVA, with spatial frequency (six levels) selected as within-subject factors, and treatment group (two levels) selected as between-subject factor, showed there was no significant differences on CS at baseline between the groups [*F*(1,168) = 2.011, *P* = 0.158] and the interaction between intervention group and spatial frequency was also not significant [*F*(5,168) = 0.214, *P* = 0.956]. Likewise, there were no significant differences between the groups in terms of AULCSF [*t*(28) = –0.720, *P* = 0.477] as demonstrated by independent-sample *t*-test.

**TABLE 1:** Clinical details of the participants.

	**Polyvinyl alcohol group**				**Atropine group**		
	**Age/Sex**	**Refraction (OD/OS)**	**logMAR VA (OU)**		**Age/Sex**	**Refraction (OD/OS)**	**logMAR VA (OU)**
*S1*	35/M	−4.00/−0.50 × 176 −4.25/−0.50 × 178	0.00	*S16*	23/F	−2.00/−1.25 × 165 −1.75/-1.25 × 5	0.00
*S2*	25/F	−2.00/−0.50 × 105 −1.75/−0.25 × 40	−0.10	*S17*	23/F	−6.00 −5.25	0.00
*S3*	25/F	−5.25 −5.00	0.00	*S18*	24/F	−3.75/−0.75 × 100 −4.25/−1.00 × 25	0.00
*S4*	24/F	−4.75 −4.50	0.00	*S19*	22/F	−1.25/−0.25 × 140 −2.25/−0.50 × 165	0.00
*S5*	22/F	−3.50 −3.00	0.00	*S20*	23/F	−3.50 −4.00/−0.75 × 160	0.00
*S6*	23/F	−4.00/−1.50 × 180 −3.75/−1.25 × 180	0.00	*S21*	25/F	−2.25/−0.75 × 55 −1.75/−1.00 × 131	0.00
*S7*	19/M	−5.25 −5.00	0.00	*S22*	20/M	−5.00/−0.50 × 180 −4.50/−0.75 × 170	0.00
*S8*	23/M	−6.00 −6.00	0.00	*S23*	23/M	−4.25/−0.25 × 60 −4.25	−0.10
*S9*	22/F	−2.00/−1.00 × 40 −1.00/−1.00 × 140	−0.10	*S24*	19/M	−4.00/−1.00 × 170 −4.00/−1.00 × 175	0.00
*S10*	25/F	−3.75 −3.25	0.00	*S25*	21/M	−3.25/−0.75 ×33 −2.50/−1.25 × 147	0.00
*S11*	19/M	−4.50/−1.00 × 90 −5.00/−1.50 × 180	0.00	*S26*	23/F	−5.50/−0.75 × 180 −5.75/−1.00 × 165	−0.10
*S12*	25/F	−4.50 −4.25	0.00	*S27*	23/F	−4.25/−1.00 × 5 −4.50/−1.00 × 165	0.00
*S13*	22/M	−2.00/−1.00 × 100 −2.25/−1.00 × 140	0.00	*S28*	25/M	−1.25 −2.25	0.00
*S14*	27/F	−3.25 −3.25	0.00	*S29*	23/M	−2.75/−0.50 × 180 −1.75	0.00
*S15*	23/M	−1.75/−0.50 × 10 −1.00/−0.25 × 150	−0.10	*S30*	24/F	−2.25/−0.50 × 90 −1.25/−0.50 × 180	0.00

*M, male; F, female; OD, oculus dexter (right eye); OS, oculus sinister (left eye); OU, oculus unati (binocular); VA, visual acuity.*

To assess whether there was any effect of atropine on CS, we firstly conducted a three-way repeated-measure ANOVA, with spatial frequency (six levels) and time (seven levels) selected as within-subject factors, and treatment group (two levels) selected as between-subject factor. We found that there was no significant difference between two groups [*F*(1,28) = 0.018, *P* = 0.895], nor significant interaction effect of treatment group and time [*F*(6,168) = 1.355, *P* = 0.254].

We then conducted a two-way repeated-measure ANOVA (two within-subject: time of measurements, seven levels; spatial frequency, six levels) on each group to figure out whether there is any time cumulative effect of atropine compared with placebo-controlled group. The two-way repeated-measure ANOVA showed that there was no significant change on CS [0.01% atropine: *F*(6,84) = 2.071, *P* = 0.114; polyvinyl alcohol: *F*(6,84) = 1.462, *P* = 0.201].

**FIGURE 3:**
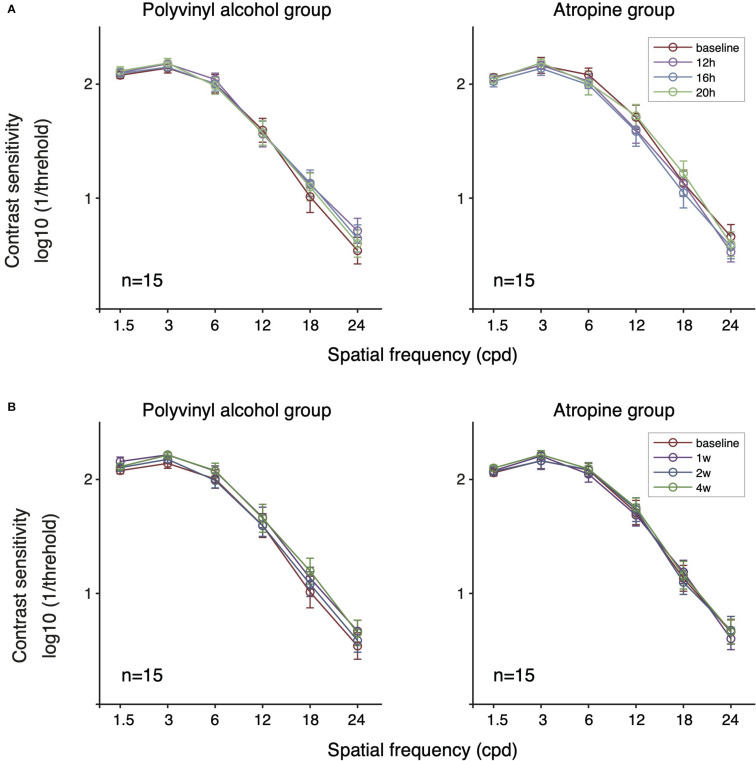
Contrast sensitivity function of the two groups following short-term **(A)** and long-term **(B)** administration of 0.01% atropine or polyvinyl alcohol. There was no significant effect of time in the polyvinyl alcohol group and the atropine group (respectively, *P* = 0.444, *P* = 0.123 at short-term; *P* = 0.094, *P* = 0.656 at long-term). There was no significant difference between the polyvinyl alcohol group and the atropine group (*P* = 0.924 and *P* = 0.724 for short- and long-term, respectively).

We next evaluated the short-term effect (within one day) of low-concentration atropine (Figure 3A). Specifically, we conducted a two-way repeated-measure ANOVA for results of baseline and 12, 16, 20 h follow-up tests for the two groups. We found that there was no significant change on CS before and after using of the eye drops for each group: 0.01% atropine: *F*(3,42) = 2.036, *P* = 0.123; polyvinyl alcohol: *F*(3,42) = 0.911, *P* = 0.444. To assess whether there is any difference between atropine group and placebo-controlled group, we then conducted a multi-factor repeated-measure ANOVA, and we found that there was no significant difference between two groups [*F*(1,28) = 0.009, *P* = 0.924], and the interaction effect of treatment group and time was also not significant [*F*(3,84) = 2.401, *P* = 0.087].

**FIGURE 4:**
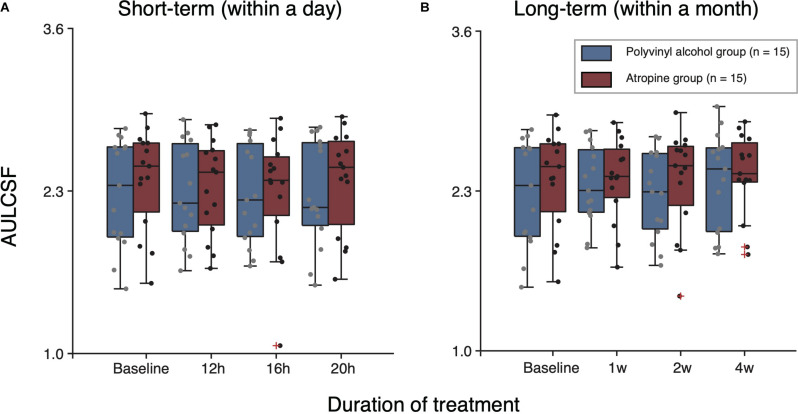
Boxplots of AULCSF following short-term **(A)** and long-term **(B)** administration of polyvinyl alcohol (Blue) and atropine (red). The black solid line within each box represents the median. The box represents the interquartile range (IQR) of the data (25th to the 75th percentile). The whisker represents 1.5 × IQR either above the third quartile or below the first quartile. Crosses represent outliers.

We conducted extra analysis for AULCSF dataset. The averaged and individual AULCSF as a function of follow-up time sessions are plotted in Figure 4A. A two-way repeated-measure ANOVA was used, which indicated no significant difference between two groups [*F*(1,28) = 0.243, *P* = 0.626], and no significant interaction effect of treatment group and time [*F*(3,84) = 0.909, *P* = 0.441]. In short, there was no significant effect of 0.01% atropine on CS in short-term within one day.

To assess whether there is long-term (e.g., a month) cumulative effect of time, we conducted a two-way repeated-measure ANOVA for results of baseline and 1, 2, and 4 weeks follow-up tests on each group (Figure 3B). The results showed there was no significant cumulative effect of 0.01% atropine on CS [atropine group: *F*(3,42) = 0.389, *P* = 0.656; polyvinyl alcohol: *F*(3,42) = 2.273, *P* = 0.094]. And multi-factor repeated-measure ANOVA suggested there was no significant difference between two groups [*F*(1,28) = 0.127, *P* = 0.724]. The interaction effect of treatment group and time was also not significant [*F*(3,84) = 1.094, *P* = 0.347]. The averaged and individual AULCSF as a function of time are plotted in Figure 4B. A multi-factor repeated-measure ANOVA was used, which revealed there was no significant difference between two groups [*F*(1,28) = 0.127, *P* = 0.724]. The interaction effect of treatment group and time was also not significant [*F*(3,84) = 1.094, *P* = 0.347]. In other word, there was no significant difference of CS found before and after using 0.01% atropine at a long-term (i.e., weeks up to one month).

## Discussion

In this study, we investigated whether the application of 0.01% atropine could influence CS in the short-term and long-term. Our results show that the effect of 0.01% atropine on adult myopes’ visual perception is minimal.

Previous studies of atropine application in myopia control have used VA as an index of visual perception and found no significant effect of 0.01% atropine on distant VA compared to placebo-controlled group as well as no significant time cumulative effect over two years (Chia et al., 2012; Yam et al., 2019; Yam et al., 2020). Although VA is the most common functional endpoint, it has limitations. VA only represents the ability of distinguishing fine details at high contrast, although objects in the real world involve a wide range of luminance and contrast levels. CS was shown to better reflect the ability of detecting and identifying objects in day-to-day experience than that of VA (Owsley and Sloane, 1987; Roark and Stringham, 2019). Moreover, CS is a much more sensitive measure than VA when diseases, degeneration or other changes occur in the visual system (Joltikov et al., 2017; Zhao et al., 2017; Alahmadi et al., 2018; Xiong et al., 2020). To illustrate, there are cases with relatively normal VA but different extents of CS deficits across various ocular pathologies (Xiong et al., 2020). Recent studies demonstrate that myopia control measures, e.g., multifocal spectacles and contact lenses, could lower participants’ low contrast VA, despite high contrast VA remaning intact (Przekoracka et al., 2020; Walline et al., 2020b). In the current study, we confirm that both short-term (e.g., within a day) and long-term (e.g., within a month) administration of 0.01% atropine didn’t have significant detrimental effect on CS in adult myopes.

Atropine is a non-selective antagonist of the muscarinic acetylcholine receptor, which is widely distributed in ocular tissues, including cornea, iris, ciliary body and ciliary muscles, epithelium of crystalline lens, retina, choroid, and sclera (Friedman et al., 1988; Gil et al., 1997; Collison et al., 2000; Qu et al., 2006; Barathi et al., 2009). Although the anti-myopia mechanism of atropine is not fully understood, recent experiments have shown that atropine may exert its myopia-protective effect mainly through muscarinic receptors on retina and sclera, stopping the remodeling and thinning of sclera and the consequent axial lengthening of the eye, even at a low concentration (Sánchez-González et al., 2020; Upadhyay and Beuerman, 2020). Moreover, topical atropine could cause pupil dilation, decrease of accommodation amplitude and change in corneal curvature, lens thickness, anterior chamber depth and vitreous chamber depth (Qu et al., 2006; Kumaran et al., 2015; Goldberg and Rucker, 2016; Yam et al., 2019; Upadhyay and Beuerman, 2020). It was proved that CS is affected by optical factors (e.g., aberration based on pupil size and intraocular forward scattering) as well as retinal and brain processing (Radhakrishnan et al., 2004; Yamaguchi et al., 2011; Kamiya et al., 2014; Karatepe et al., 2017). In animal experiments in myopia control, CS at low spatial frequency was enhanced after the administration of atropine (Diether and Schaeffel, 1999; Schmucker and Schaeffel, 2006). Schmucker and Schaeffel (2006) thought the increase of CS in mice was possibly due to the dilatation of the pupil with atropine and the brighter retinal image, despite the larger pupil also resulting in a decline in optical quality of retinal image. As a treatment of amblyopia, it has been found that the CS of amblyopic patients improves after the administration of atropine by suppressing fellow eye and meliorating VA of amblyopia eye (Menon et al., 2008; DeSantis, 2014). The anti-myopia mechanism of atropine is totally different from the rationale for amblyopia therapy. We were interested in whether low-concentration atropine could influence myopes’ CS. Our results show that no significant differences existed in CSF before and after both short- and long-term administration of 0.01% atropine in adult myopes.

The results agrees with Anders et al.’s (2019) study, which showed there was no pronounced impact of 0.01% atropine on retinal processing, as reflected by the pattern electroretinogram (PERG). Moreover, Khanal et al. (2019) found that atropine may exert its anti-myopia effect mainly through affecting the responses to myopia defocus in peripheral retinal instead of central retinal as demonstrated by the global flash multifocal electroretinogram (gmfERG). And there was no significant change on VA after the application of 0.01% atropine (Chia et al., 2012; Yam et al., 2019; Yam et al., 2020). Our study, together with these previous reports, suggests that atropine might produce minimal effect on macular visual functions.

Another possible reason for the minimal effect is that the biochemical and structural changes in ocular system caused by one-month administration of 0.01% atropine was too slight to be detected. Yet there were significant dose-related effects of atropine in axial length, accommodation and pupil diameter (Chia et al., 2012; Yam et al., 2019; Fu et al., 2020; Li et al., 2020; Yam et al., 2020). In the LAMP study (Yam et al., 2019), the change of mesopic pupil size was only 0.23 mm in 0.01% atropine group compared to 0.43 and 0.58 mm in 0.025 and 0.05% groups after using eye drops for a year. Hence, a future study should investigate whether higher concentration (e.g., 0.025, 0.05%) of atropine affects CS.

In conclusion, our results indicate that 0.01% atropine has minimal deleterious effect on patient’s CS. It should be noted that our research was conducted on adult myopia, while topical atropine is mainly used by preschool and school-age pediatric, who are at a stage of rapid myopia progression (Lin et al., 2018). A reduced visual perception (e.g., VA, CS) would be a disadvantage for kids, as it might produce amblyopia (McKee et al., 2003; Levi, 2020). It is an interesting question that whether a myopia control strategy that slightly decreases patients’ visual perception would produce a worse or better myopia control effect than those strategies that didn’t change patients’ visual perception (Lau et al., 2020; Vincent et al., 2020). We have no clear answer to this question at this stage, since our knowledge of the change of visual functions following myopia control is limited. Further investigations on children myopia are needed to better show the effect of 0.01% atropine on their perception. Also, we only apply the atropine for one month. According to the results in Figure 4, there seem to be a trend, albeit non-significant, of a small reduction in sensitivity. It would be interesting to evaluate whether longer-term use (e.g., a year or longer) of atropine would affect the visual functions, since myopia control measures are typically prescribed until myopia progression is slowed down in late adolescent period (i.e., 15–18 years old) (Zadnik et al., 2015; Wu et al., 2019).

## Data Availability Statement

The original contributions presented in the study are included in the article, further inquiries can be directed to the corresponding authors.

## Ethics Statement

The studies involving human participants were reviewed and approved by the Ethics Committee of Affiliated eye hospital, Wenzhou Medical University. The participants provided their written informed consent to participate in this study.

## Author Contributions

ZC, JM, YW, and JZ conceived the experiments. ZC, JM, SC, and RZ performed the experiments. ZC, JM, and JZ analyzed the data and interpreted the data. ZC, YW, and JZ wrote the manuscript. All authors contributed to manuscript revision, read and approved the submitted version.

## Conflict of Interest

The authors declare that the research was conducted in the absence of any commercial or financial relationships that could be construed as a potential conflict of interest.
